# DCE-MRI of locally-advanced carcinoma of the uterine cervix: Tofts analysis versus non-model-based analyses

**DOI:** 10.1186/s13014-020-01526-2

**Published:** 2020-04-15

**Authors:** Kjersti V. Lund, Trude G. Simonsen, Gunnar B. Kristensen, Einar K. Rofstad

**Affiliations:** 1 Department of Radiation Biology, Institute for Cancer Research, Oslo University Hospital, Oslo, Norway; 2 Department of Radiology and Nuclear Medicine, Oslo University Hospital, Oslo, Norway; 3 Department of Gynecological Cancer, Oslo University Hospital, Oslo, Norway; 4 Institute for Cancer Genetics and Informatics, Oslo University Hospital, Oslo, Norway

**Keywords:** Cervical carcinoma, Biomarkers, DCE-MRI, Tofts pharmacokinetic model

## Abstract

**Background:**

Dynamic contrast-enhanced magnetic resonance imaging (DCE-MRI) may provide biomarkers of the outcome of locally-advanced cervical carcinoma (LACC). There is, however, no agreement on how DCE-MR recordings should be analyzed. Previously, we have analyzed DCE-MRI data of LACC using non-model-based strategies. In the current study, we analyzed DCE-MRI data of LACC using the Tofts pharmacokinetic model, and the biomarkers derived from this analysis were compared with those derived from the non-model-based analyses.

**Methods:**

Eighty LACC patients given cisplatin-based chemoradiotherapy with curative intent were included in the study. Treatment outcome was recorded as disease-free survival (DFS) and overall survival (OS). DCE-MRI series were analyzed voxelwise to produce *K*^trans^ and *v*_e_ frequency distributions, and ROC analysis was used to identify the parameters of the frequency distributions having the greatest potential as biomarkers. The prognostic power of these parameters was compared with that of the non-model-based parameters LETV (low-enhancing tumor volume) and TVIS (tumor volume with increasing signal).

**Results:**

Poor DFS and OS were associated with low values of *K*^trans^, whereas there was no association between treatment outcome and *v*_e_. The *K*^trans^ parameters having the greatest prognostic value were p35-*K*^trans^ (the *K*^trans^ value at the 35 percentile of a frequency distribution) and RV-*K*^trans^ (the tumor subvolume with *K*^trans^ values below 0.13 min^− 1^). Multivariate analysis including clinical parameters and p35-*K*^trans^ or RV-*K*^trans^ revealed that RV-*K*^trans^ was the only independent prognostic factor of DFS and OS. There were significant correlations between RV-*K*^trans^ and LETV and between RV-*K*^trans^ and TVIS, and the prognostic power of RV-*K*^trans^ was similar to that of LETV and TVIS.

**Conclusions:**

Biomarkers of the outcome of LACC can be provided by analyzing DCE-MRI series using the Tofts pharmacokinetic model. However, these biomarkers do not appear to have greater prognostic value than biomarkers determined by non-model-based analyses.

## Background

Tumor hypoxia is a major cause of treatment failure in patients with locally-advanced cervical carcinoma (LACC) given cisplatin-based chemoradiotherapy [1–3]. The outcome of LACC may be improved by personalizing the treatment, and personalized therapy of LACC requires novel biomarkers of treatment outcome [4]. Dynamic contrast-enhanced magnetic resonance imaging (DCE-MRI) may provide information on the extent of hypoxia in LACC [4–7], and the outcome of LACC has been shown to be associated with parameters derived from DCE-MRI data [8–12]. However, these associations are fairly weak, and a sound DCE-MRI strategy for predicting the outcome of LACC has yet to be developed.

We have analyzed DCE-MRI data of LACC voxelvise and identified two biomarkers of disease-free survival (DFS) and overall survival (OS): low-enhancing tumor volume (LETV) [13] and tumor volume with increasing signal intensity (TVIS) [14]. LETV refers to the tumor volume showing low contrast enhancement during the first 60 s of the recordings, and TVIS represents the tumor volume showing increasing contrast enhancement during a 6-min-long interval in the late-phase of the DCE-MRI series [13, 14]. The calculation of LETV and TVIS was not based on any pharmacokinetic model.

Two pharmacokinetic models are used frequently to analyze clinical DCE-MRI series: the Brix model [15, 16] and the Tofts model [17, 18]. These models are based on physiological properties of tumors, and consequently, it may be preferable to analyze DCE-MR recordings of tumors using model-based strategies rather than non-model-based strategies [19]. In an earlier study of LACC, we compared the prognostic power of the parameters of the Brix model with that of LETV and TVIS and concluded that biomarkers derived from Brix analysis are not superior to LETV and TVIS [20].

In the present investigation, the DCE-MR recordings of the LACC patients included in our studies of LETV and TVIS were analyzed using the Tofts model. The Tofts model has the advantage to the Brix model that the analysis can be based on the concentration of contrast agent in the tumor tissue rather than signal intensity [15–18]. The objective of the investigation was to determine whether the parameters of the Tofts model may be clinically useful biomarkers of the outcome of LACC and to compare the prognostic value of these biomarkers with that of LETV and TVIS.

## Materials and methods

### Patients

Eighty patients with untreated LACC (FIGO stage IB through IVA) admitted to the Norwegian Radium Hospital were included in the study. The demographics of the patients have been described earlier [13, 14]. After DCE-MRI, the patients were treated with concurrent cisplatin-based chemoradiotherapy with curative intent. Briefly, external beam radiation therapy was given in 25 fractions during a period of 5 weeks to a total dose of 50 Gy to the primary tumor, parametria, and ajacent pelvic wall and 45 Gy to the rest of the pelvic region. In addition, 5–6 fractions of intracavitary brachytherapy with a dose of 4.2 Gy per fraction were given to Point A. Chemotherapy with cisplatin (40 mg/m^2^) was given weekly with a maximum of 6 courses during the radiation therapy period.

The primary endpoints were DFS, defined as the time from diagnosis to local or distant relapse or death, and OS, defined as the time from diagnosis to death. The study was approved by the regional committee of medical research ethics in southern Norway and was carried out in accordance with the Declaration of Helsinki. Informed consent was obtained from all patients.

### MRI

The MRI protocol has been described in detail elsewhere [13]. Briefly, the pelvis was scanned with axial *T*_2_-weighted fast spin echo sequences, and tumor volume and lymph node status were assessed by examining the *T*_2_-weighted images in the dicom viewer Osirix. A gradient echo sequence was used to generate axial proton density-weighted images prior to and after DCE-MRI. DCE-MRI was conducted by using an axial *T*_1_-weighted spoiled gradient recalled sequence [repetition time: 160 ms; echo time: 3.5 ms; flip angle: 90°; number of excitations: 1; field of view: 20 × 20 cm^2^; image matrix: 256 × 256; slice thickness: 5 mm; slice spacing: 6 mm; voxel size: 0.78 × 0.78 × 5.0 mm^3^; temporal resolution: 29 s; sampling time: 10 min]. Three precontrast images were acquired, and contrast agent was injected manually in a bolus dose of 0.1 mmol/kg Gd-DTPA during a period of ~ 3 s. To enable calculation of the concentration of Gd-DTPA in the tumor tissue, a calibration tube with two chambers, one filled with saline and the other with a 0.5-mmol Gd-DTPA solution, was inserted into the vagina prior to the imaging. The tube was positioned as close to the tumor as possible, and acceptable positioning was verified by MRI.

### Image analysis

Voxel-by-voxel analysis of DCE-MR images was conducted by using software developed in Matlab. Minor movements of the tumor tissue during image acquisition were corrected for by coordinate mapping. To avoid significant influence of body cavities and tumor necrosis, voxels showing signal intensities in *T*_2_-weighted images consistent with the presence of air or water were excluded from analysis. Gd-DTPA concentrations were calculated from signal intensities using the method of Hittmair et al. [21], as described in detail elsewhere [22].

Plots of Gd-DTPA concentration versus time after contrast injection were generated, and the Levenberg-Marquardt least squares minimization method was used to fit curves to the data, using the standard Tofts equation [18]:

$$ {C}_{\mathrm{t}}(T)={K}^{\mathrm{t}\mathrm{rans}}\bullet {\int}_0^T{C}_{\mathrm{p}}(t)\bullet {e}^{-\frac{K^{\mathrm{t}\mathrm{rans}}\bullet \left(T-t\right)}{V_e}}\  dt. $$

Here, *C*_t_(*T*) is the tumor tissue concentration of Gd-DTPA at time *T*, *K*^trans^ is the volume transfer constant of Gd-DTPA, *v*_e_ is the fractional distribution volume of Gd-DTPA in the tumor tissue, and *C*_p_(*t*) is the arterial input function:

$$ {C}_{\mathrm{p}}(t)=\mathrm{A}\bullet {e}^{-\mathrm{B}t}+\mathrm{C}\bullet {e}^{-\mathrm{D}t}, $$

where *C*_p_(*t*) is the plasma concentration of Gd-DTPA at time *t*, and A = 5.10 mM, B = 14.2 s^− 1^, C = 0.99 mM, and D = 0.159 s^− 1^. Frequency distributions and parametric images of *K*^trans^ and *v*_e_ were generated by using SigmaPlot software.

### Statistical analysis

Because standard first-line treatment fails in 60–70% of the patients diagnosed with LACC [23], associations between Tofts parameters and DFS or OS were studied by dividing the patient cohort into two groups consisting of one-third and two-thirds of the patients [13]. Thus, the DFS and OS of the 26 patients with the lowest *K*^trans^ values were compared with the DFS and OS of the 54 patients with the highest *K*^trans^ values, and similar comparisons were carried out for *v*_e_. Kaplan–Meier curves were compared by using the log-rank test. Univariate and multivariate Cox proportional hazard analyses were used to evaluate the prognostic power of clinical and DCE-MRI–derived parameters. The Spearman rank order correlation test was used to search for correlations between parameters. Probability values of *p* < 0.05 were considered significant.

## Results

**Fig. 1:**
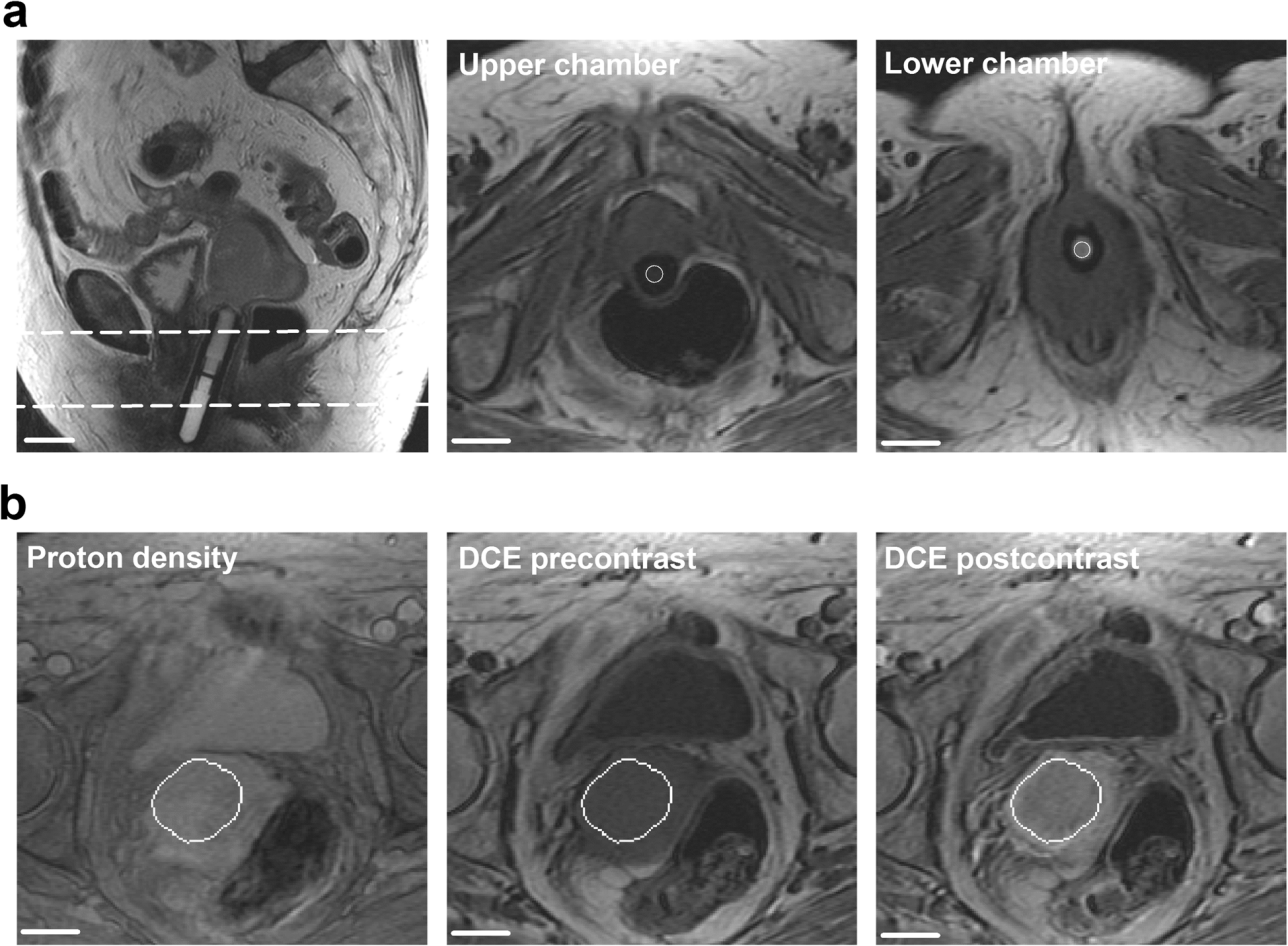
Representative anatomical MR images. **a** A sagittal and two axial *T*_1_-weighted scans showing the signal intensities of the two-chamber calibration tube. The dashed horizontal lines indicate the positions of the axial scans. **b** A proton density-weighted image, a precontrast *T*_1_-weighted image, and a postcontrast *T*_1_-weighted image showing the signal intensities of the tumor tissue. Scale bars: 2 cm

Relevant anatomical MR images of a representative LACC patient are presented in Fig. 1. The position and signal intensities of the calibration tube are illustrated in a sagittal and two axial precontrast *T*_1_-weighted images (Fig. 1a), and a proton density-weighted image, a precontrast *T*_1_-weighted image, and a postcontrast *T*_1_-weighted image are shown to illustrate the signal intensities of the tumor tissue (Fig. 1b).

**Fig. 2:**
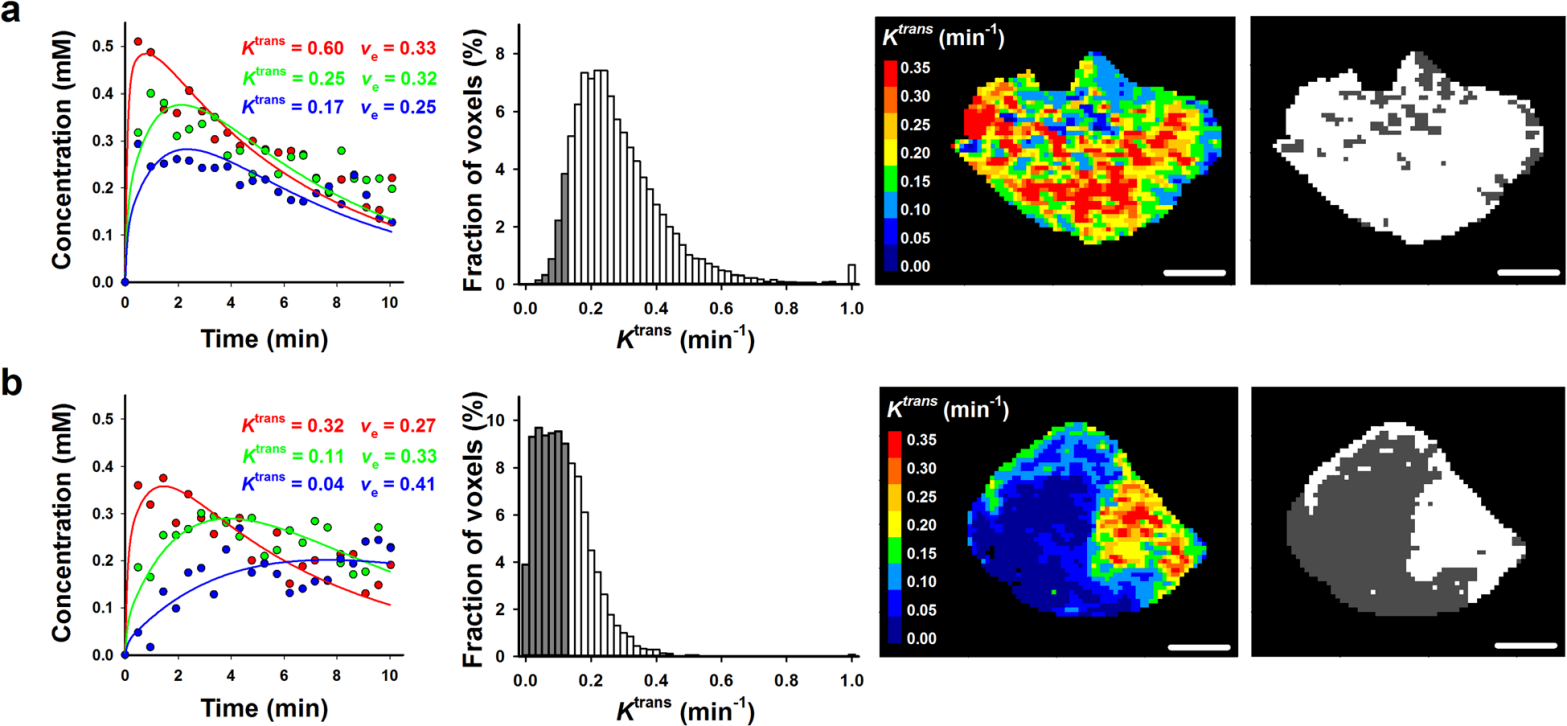
Representative *K*^trans^ data. Single-voxel plots of Gd-DTPA concentration versus time after contrast injection, *K*^trans^ frequency distribution, parametric *K*^trans^ image, and binary *K*^trans^ image of **a** a high-enhancing tumor and **b** a low-enhancing tumor. The dark grey regions in the frequency distributions and binary images represent RV-*K*^trans^. Scale bars: 1 cm

Tumor signal enhancement after Gd-DTPA administration differed substantially among individual patients, and Fig. 2 presents *K*^trans^ data for two representative patients, one with a high-enhancing tumor (Fig. 2a) and the other with a low-enhancing tumor (Fig. 2b). The Tofts model was seen to be well suited for analyzing the DCE-MRI series. Single-voxel plots of Gd-DTPA concentration versus time were well fitted by the model, and the numeric values of *K*^trans^ were generally higher in high-enhancing than in low-enhancing tumors. However, the intratumor heterogeneity in *K*^trans^ was substantial, as illustrated by the *K*^trans^ frequency distributions and parametric maps in Fig. 2.

**Fig. 3:**
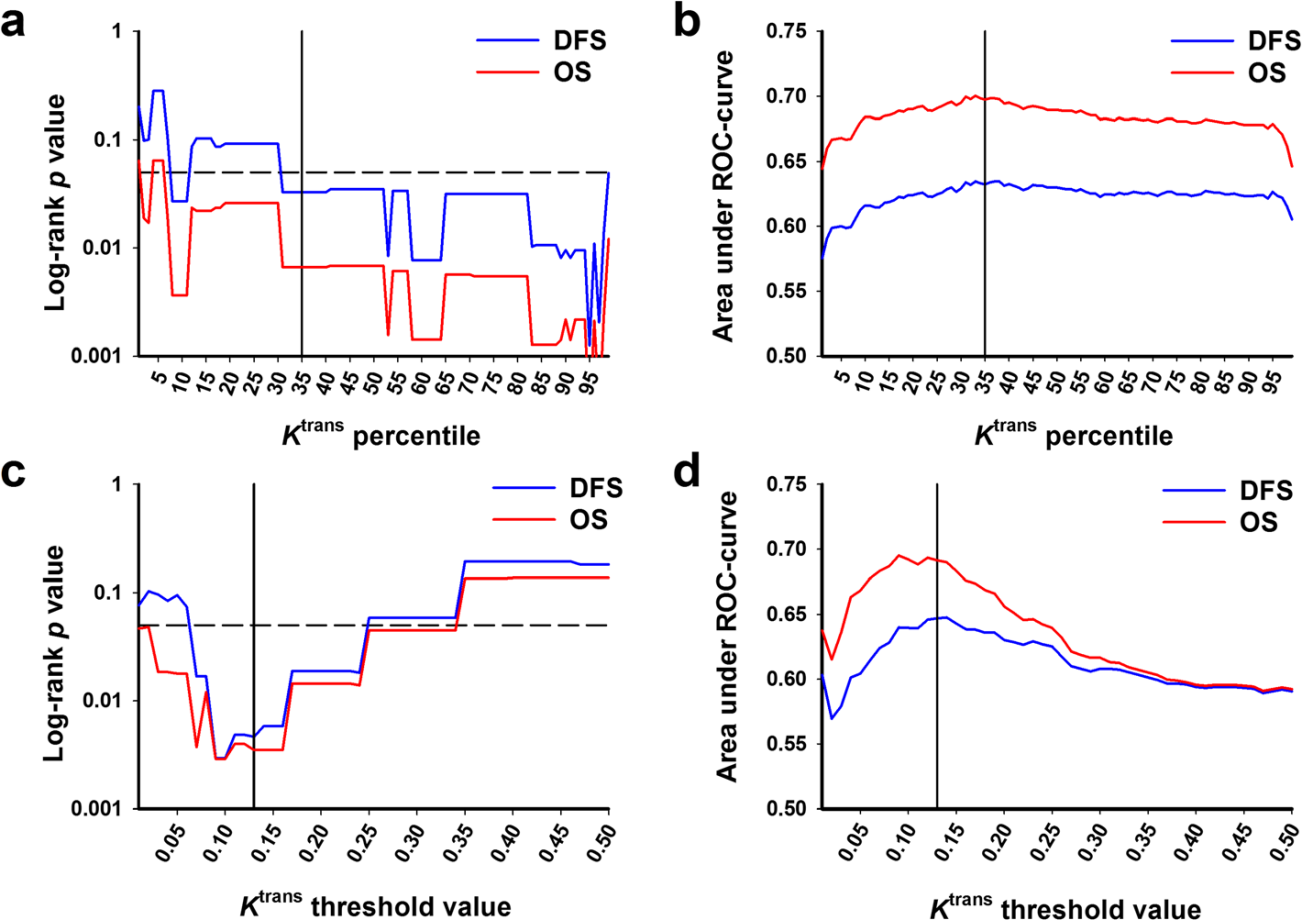
Log-rank and ROC analyses. The values of *K*^trans^ at each percentile of the frequency distributions and the tumor volumes (RV-*K*^trans^) with *K*^trans^ values below a wide range of threshold values were calculated for the 80 tumors included in the study, and for each *K*^trans^ percentile and each *K*^trans^ threshold value, the outcome of the patients with high values of *K*^trans^ or RV-*K*^trans^ was compared with the outcome of those with low values, using the log-rank test with DFS and OS as endpoints. ROC analysis was carried out to indentify the *K*^trans^ percentile and *K*^trans^ threshold value with the highest discriminative power. **a** Log-rank *p* value versus *K*^trans^ percentile. **b** Area under ROC-curve versus *K*^trans^ percentile. **c** Log-rank *p* value versus *K*^trans^ threshold value. **d** Area under ROC-curve versus *K*^trans^ threshold value

Detailed analyses of the *K*^trans^ frequency distributions were carried out to investigate whether *K*^trans^ may provide valid prognostic information on DFS and OS. First, the *K*^trans^ values at each integer percentile of the frequency distributions were determined, and a log-rank test was conducted for each percentile to examine whether the *K*^trans^ values at any of the percentiles could discriminate between the two patient groups. Plots of the log-rank *p* value versus *K*^trans^ percentile were established, and the plots revealed that the *K*^trans^ values at percentiles above 30 provided *p* values < 0.05 for both DFS and OS (Fig. 3a). Receiver operating characteristic (ROC) analysis was carried out to determine the optimal *K*^trans^ percentile. The area under the ROC-curve is plotted versus *K*^trans^ percentile in Fig. 3b, and the optimal percentile, corresponding the largest area under the ROC-curve, was found to be the 35 percentile for DFS as well as OS. The *K*^trans^ value at this percentile was termed 35p-*K*^trans^.

Next, we calculated the number of voxels and the corresponding tumor volumes with *K*^trans^ values below a wide range of threshold values up to 0.50 min^− 1^, using increments of 0.0025 min^− 1^. A log-rank test was carried out for each threshold value to investigate whether the tumor volumes at any of the threshold values could discriminate between the two patient groups. Plots of the log-rank *p* value versus *K*^trans^ threshold value were generated for DFS and OS, and these plots showed that *K*^trans^ threshold values between 0.07 min^− 1^ and 0.24 min^− 1^ provided *p* values < 0.05 for both endpoints (Fig. 3c). ROC analysis revealed that the optimal *K*^trans^ threshold value was 0.13 min^− 1^, regardless of whether DFS or OS was considered (Fig. 3d). The tumor volume with *K*^trans^ values below 0.13 min^− 1^ was presumed to represent the risk volume (RV) and was termed RV-*K*^trans^.

Binary tumor maps showing the distribution of voxels with *K*^trans^ values above or below 0.13 min^− 1^ are included in Fig. 2. In general, the voxels constituting RV-*K*^trans^ were contiguous in tumors having a large RV-*K*^trans^ (Fig. 2b).

**Fig. 4:**
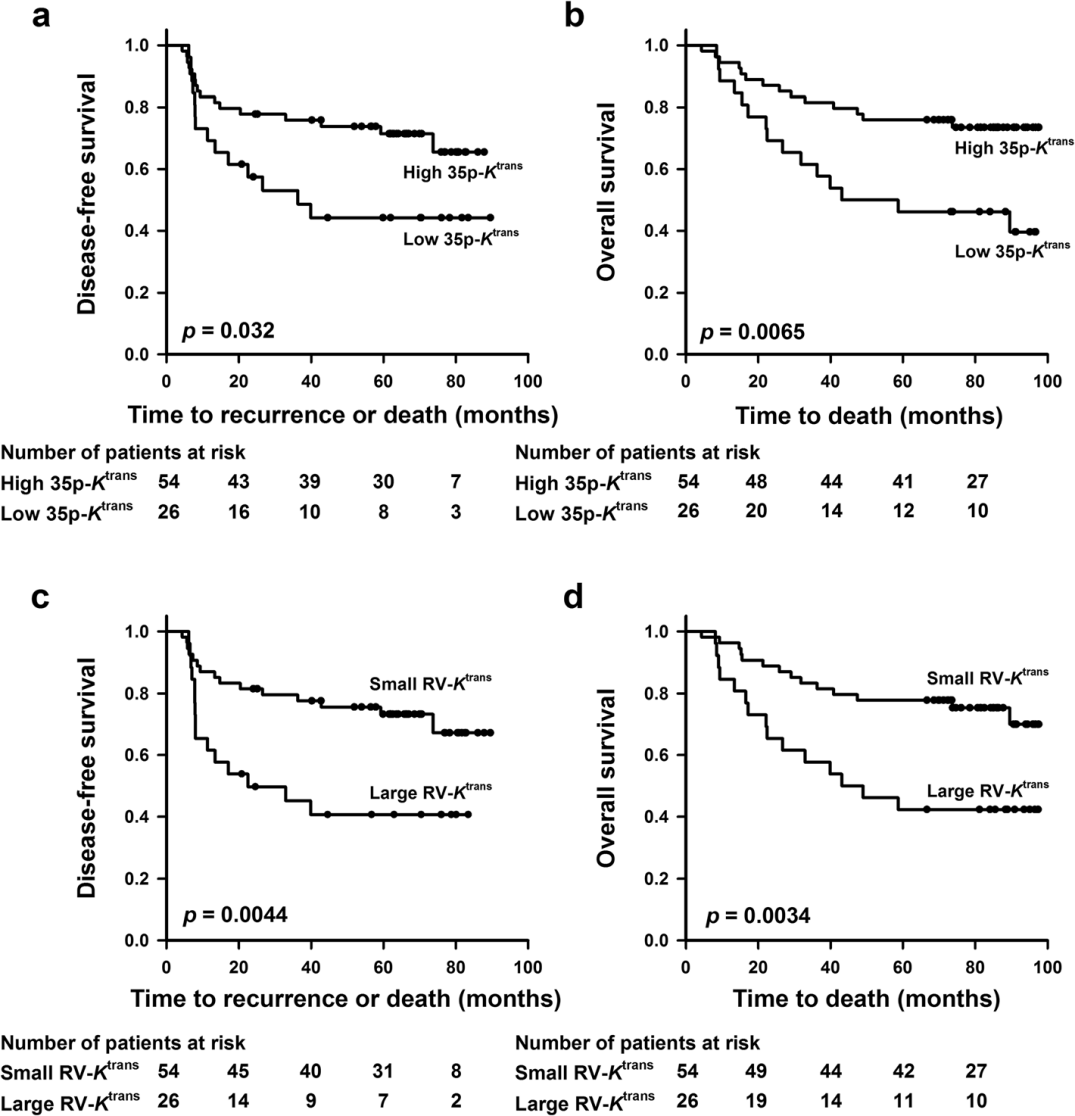
Treatment outcome. Kaplan–Meier curves for DFS and OS of LACC patients stratified by 35p-*K*^trans^**a,b** and RV-*K*^trans^**c,d**. *p* values: log-rank test

Kaplan–Meier plots for DFS and OS based on 35p-*K*^trans^ and RV-*K*^trans^ are presented in Fig. 4. The patients with low *K*^trans^ values did worse than those with high *K*^trans^ values. The 5-year survival rates of the patients with low 35p-*K*^trans^ values and those with high 35p-*K*^trans^ values were 44 and 72%, respectively (DFS: *p* = 0.032) and 46 and 76%, respectively (OS: *p* = 0.0065). Similarly, the patients with large RV-*K*^trans^ and those with small RV-*K*^trans^ showed 5-year DFS rates of 41 and 73%, respectively (*p* = 0.0044) and 5-year OS rates of 42 and 78%, respectively (*p* = 0.0034).

Univariate Cox regression analysis showed that 35p-*K*^trans^ and RV-*K*^trans^ had significant impact on OS, and that RV-*K*^trans^ but not 35p-*K*^trans^ had significant impact on DFS (Table 1). A similar analysis of clinical parameters revealed that DFS and OS were influenced significantly by tumor volume and FIGO stage, but not by lymph node status, tumor histology, and patient age (Table 1). Furthermore, RV-*K*^trans^ was found to be the only independent prognostic factor of DFS and OS in multivariate Cox regression analysis involving tumor volume, FIGO stage, lymph node status, and 35p-*K*^trans^ or RV-*K*^trans^ (Table 1).

**Table 1:** Cox regression analysis of clinical and DCE-MRI–derived parameters

	Univariate		Multivariate			
	Disease-free survival	Overall survival	Disease-free survival		Overall survival	
	*p* value	*p* value	*p* values^a^		*p* values^a^	
Tumor volume	**0.026**	**0.019**	0.49	0.69	0.18	0.76
FIGO stage	**0.0057**	**0.017**	0.065	0.055	0.099	0.091
Lymph node status	0.13	0.36	0.73	0.84	0.81	0.60
Tumor histology	0.30	0.31	–	–	–	–
Patient age	0.30	0.17	–	–	–	–
35p-*K*^trans^	0.15	**0.047**	0.20	–	0.057	–
RV-*K*^trans^	**0.00033**	**< 0.00001**	–	**0.026**	–	**0.0015**

Values of *p* < 0.05 are highlighted in bold

^a^*p* values refer to multivariate regression analyses including tumor volume, FIGO stage, lymph node status, and either

35p-*K*^trans^ or RV-*K*^trans^

35p-*K*^trans^, the value of *K*^trans^ at the 35 percentile of the *K*^trans^ frequency distribution of a tumor

RV-*K*^trans^, the tumor subvolume with *K*^trans^ values < 0.13 min^−1^

**Fig. 5:**
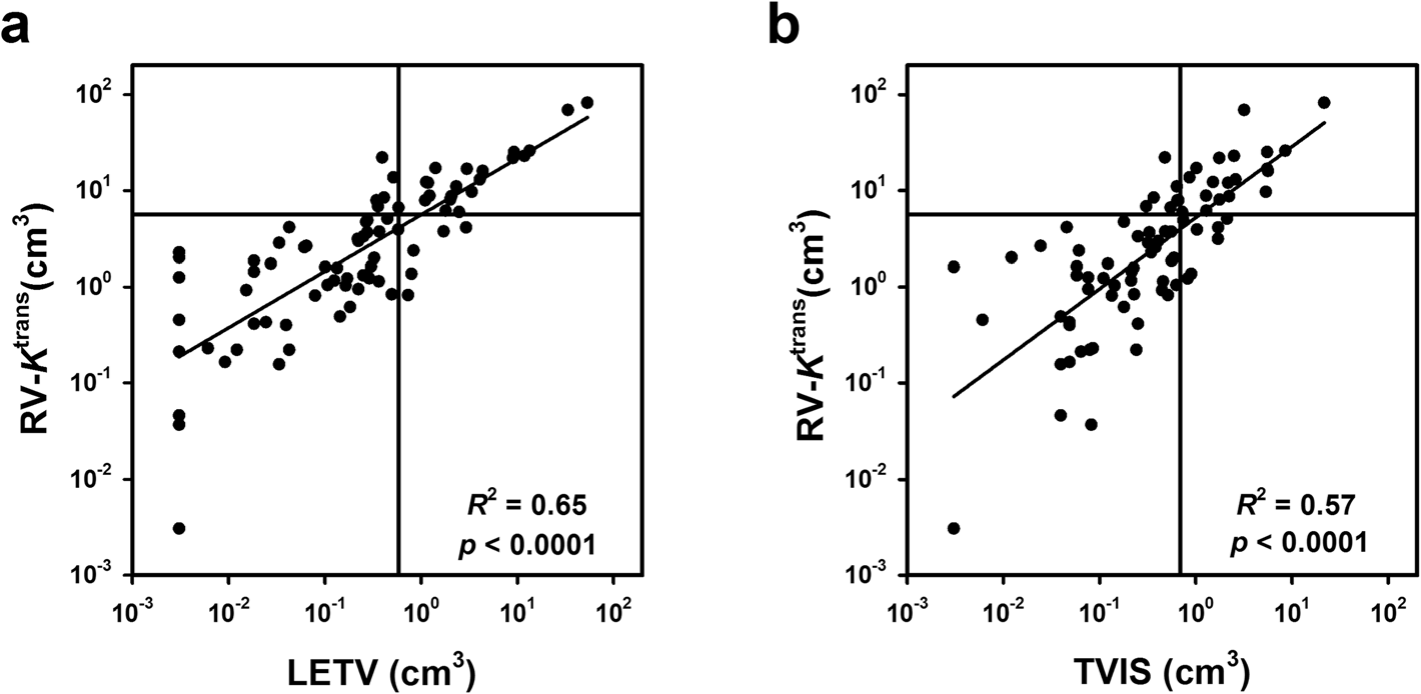
Model-based versus non-model-based RVs. Log scale plots of RV-*K*^trans^ versus LETV **a** and TVIS **b** for patients with LACC. The patient cohort was divided into two groups consisting of one-third and two-thirds of the patients, and the discrimination levels are indicated by horizontal lines for RV-*K*^trans^ and by vertical lines for LETV and TVIS. Points: individual tumors. Curves: linear regression. *p* values: Spearman rank order correlation test

The DCE-MR recordings of the patients included in this study have been subjected to non-model-based analyses previously, and LETV and TVIS were identified as independent prognostic factors [13, 14]. LETV and TVIS represent tumor RVs, and plots of RV-*K*^trans^ versus LETV and TVIS revealed strong correlations between the RV derived from the Tofts analysis and the RVs derived from the non-model-based analyses (*p* < 0.0001; Fig. 5). The horizontal and vertical lines in Fig. 5 represent the border between small and large RVs, and show that the majority of the patients were stratified into the same risk group by the model-based and non-model-based RVs.

By subjecting the *v*_e_ frequency distributions of the tumors to analyses similar to those described above for the *K*^trans^ frequency distributions, it was seen that *v*_e_ did not provide valid prognostic information on DFS or OS. Parametric *v*_e_ images and *v*_e_ frequency distributions of the tumors used as examples in Fig. 2, and plots of log-rank *p* value versus *v*_e_ percentile and log-rank *p* value versus *v*_e_ threshold value are presented in Additional file 1: Figure S1.

## Discussion

Imaging plays an important role in the diagnostics, radiation therapy planning, and treatment monitoring of LACC [4]. In addition to MRI, positron emission tomography (PET)/computed tomography (CT) has shown promise for hypoxia imaging. Current studies are investigating the potential of several PET hypoxia tracers, including ^18^F-fluoromisonidazole, ^18^F-fluoroerythronitroimidazole, ^18^F-fluoroazomycin-arabinoside, and ^60^Cu-diacetyl-bis(N-methylthiosemicarbazone), and interesting observations have been reported [24]. It has also been hypothesized that ^18^F-fluorodeoxyglucose (^18^F-FDG) may be a clinically useful surrogate hypoxia tracer [24]. If this hypo-thesis proves to be valid, ^18^F-FDG PET/CT may turn out be a powerful imaging modality for LACC, since ^18^F-FDG PET/CT already is used successfully to detect pathological lymph nodes and high-risk radiation therapy targets [25, 26].

The potential of DCE-MRI as an imaging modality for providing hypoxia-associated biomarkers of the outcome of LACC was investigated in the study reported in this communication. DCE-MRI series of LACC were analyzed by using the Tofts pharmacokinetic model, and the prognostic power of biomarkers derived from the Tofts analysis was compared with that of biomarkers derived from non-model-based analyses. Tofts analysis has the advantage to non-model-based analyses that it is based on the physiology of the imaged tumor, but to utilize this advantage in full, information on the concentration of contrast agent in the tissue is required. This information was provided in the present study by using a two-chamber vaginal calibration tube.

Significant associations were found between p35-*K*^trans^ or RV-*K*^trans^ on the one hand side and DFS or OS on the other, as revealed by univariate Cox regression analysis. Multivariate analysis including clinical parameters and p35-*K*^trans^ or RV-*K*^trans^ showed that RV-*K*^trans^ was a strong independent prognostic factor of DFS and OS, whereas p35-*K*^trans^, tumor volume, FIGO stage, and lymph node status were not independent prognostic factors. These observations suggest that Tofts analysis of DCE-MRI data can provide biomarkers of the outcome of LACC. Moreover, the prognostic power of RV-*K*^trans^ is strong compared with that of the *K*^trans^ value at any percentile of the *K*^trans^ frequency distribution.

Poor treatment outcome was associated with low values of *K*^trans^ and, consequently, large values of RV-*K*^trans^. Clinical investigations have revealed that low *K*^trans^ values reflect poor vascularization and blood supply in LACC [9, 27, 28]. Preclinical studies of human cervical carcinoma xenografts have shown strong inverse correlations between median *K*^trans^ and fraction of hypoxic tumor tissue, regardless of whether radiobiological or pimonidazole-based immunohistochemical assays were used to quantify tumor hypoxia [5, 29–31]. Therefore, it is reasonable to assume that RV-*K*^trans^ mirrors the hypoxic subvolume in tumors of the uterine cervix. This assumption is consistent with investigations having shown that poor outcome of LACC is associated with low oxygen tension in the primary tumor, as measured with polarographic Eppendorf pO_2_ electrodes [1–3].

RV-*K*^trans^ correlated strongly with the non-model-based DCE-MRI–derived parameters LETV and TVIS, and the vast majority of the patients were stratified into the same risk group by RV-*K*^trans^, LETV, and TVIS. Furthermore, the RV-*K*^trans^-based Kaplan-Meier plots for DFS and OS reported here were similar to the LETV-based and TVIS-based Kaplan-Meier plots reported previously [13, 14]. Consequently, the prognostic power of biomarkers identified by Tofts analysis does not appear to be stronger than that of biomarkers identified by non-model-based analyses.

RV-*K*^trans^, LETV, and TVIS have specific disadvantages and advantages as biomarkers of LACC. RV-*K*^trans^ has the disadvantage that an arterial input function and long scanning times are needed for its assessment. The advantage of RV-*K*^trans^ is that it is related to the extent of tumor hypoxia. The assessment of LETV requires signal intensity-dependent threshold values [13], and because signal intensities vary depending upon MR protocol and scanner, radiology departments have to establish their own threshold values. The advantage of LETV is that short scanning times of only 1–2 min are needed, and this advantage is of significant importance because scanning time is a major limiting factor in many centers. Similar to the calculation of RV-*K*^trans^, the calculation of TVIS requires long scanning times, but the advantage of TVIS is that the calculation is independent of signal intensity, thus facilitating comparisons of results across institutions.

The current investigation has some limitations. First, *T*_2_-weighted images were used to delineate tumors and to exclude image regions influenced by body cavities and tumor necrosis, and despite the fact that these tasks were carried out by two experienced radiologists, there may have been some uncertainties in the assessment of the regions of interest. Second, the MR images were recorded several years ago when the spatial and temporal resolutions of the recordings were poorer than today’s standard due to scanner limitations. Third, the Tofts analysis was performed by using a population-based arterial input function rather than individual arterial input functions. Fourth, only 80 patients were included in the study, and consequently, it requires validation in an independent patient cohort. Despite these limitations, important conclusions can be drawn from our investigation.

## Conclusions

By subjecting DCE-MRI series of LACC to pharmacokinetic analysis using the Tofts model, prognostic factors independent of well-established clinical prognostic factors can be provided. Furthermore, RV-*K*^trans^ has stronger prognostic power than the *K*^trans^ value at any percentile of the *K*^trans^ frequency distribution. However, the prognostic value of RV-*K*^trans^ is not necessarily superior to the prognostic value of RVs assessed by non-model-based analyses.

## Supplementary information

**Additional file 1.** DCE-MRI of locally-advanced carcinoma of the uterine cervix: Tofts analysis versus non-model-based analyses

## Data Availability

The datasets used and/or analyzed during the current study are available from the corresponding author on reasonable requests.

## Abbreviations

DCE-MRI: Dynamic contrast-enhanced magnetic resonance imaging
DFS: Disease-free survival
Gd-DTPA: Gadolinium diethylenetriamine pentaacetic acid
LACC: Locally-advanced cervical carcinoma
LETV: Low-enhancing tumor volume
OS: Overall suvival
ROC: Receiver operating characteristic
RV: Risk volume
TVIS: Tumor volume with increasing signal intensity

## References

[1] Höckel M, Knoop C, Schlenger K, Vorndran B, Baussmann E, Mitze M (1993). Intratumoral pO_2_ predicts survival in advanced cancer of the uterine cervix. Radiother Oncol.

[2] Fyles AW, Milosevic M, Wong R, Kavanagh MC, Pintilie M, Sun A (1998). Oxygenation predicts radiation response and survival in patients with cervix cancer. Radiother Oncol.

[3] Sundfør K, Lyng H, Tropé CG, Rofstad EK (2000). Treatment outcome in advanced squamous cell carcinoma of the uterine cervix: relationships to pretreatment tumor oxygenation and vascularization. Radiother Oncol.

[4] Barwick TD, Taylor A, Rockall A (2013). Functional imaging to predict tumor response in locally advanced cervical cancer. Curr Oncol Rep.

[5] Ellingsen C, Hompland T, Galappathi K, Mathiesen B, Rofstad EK (2014). DCE-MRI of the hypoxic fraction, radioresponsiveness, and metastatic propensity of cervical carcinoma xenografts. Radiother Oncol.

[6] Cooper RA, Carrington BM, Loncaster JA, Todd SM, Davidson SE, Logue JP (2000). Tumour oxygenation levels correlate with dynamic contrast-enhanced magnetic resonance imaging parameters in carcinoma of the cervix. Radiother Oncol.

[7] Lyng H, Vorren AO, Sundfør K, Taksdal I, Lien HH, Kaalhus O (2001). Assessment of tumor oxygenation in human cervical carcinoma by use of dynamic Gd-DTPA-enhanced MR imaging. J Magn Reson Imaging.

[8] Hawighorst H, Weikel W, Knapstein PG, Knopp MV, Zuna I, Schönberg SO (1998). Angiogenic activity of cervical carcinoma: assessment by functional magnetic resonance imaging-based parameters and a histomorphological approach in correlation with disease outcome. Clin Cancer Res.

[9] Zahra MA, Hollingsworth KG, Sala E, Lomas DJ, Tan LT (2007). Dynamic contrast-enhanced MRI as a predictor of tumour response to radiotherapy. Lancet Oncol.

[10] Yuh WTC, Mayr NA, Jarjoura D, Wu D, Grecula JC, Lo SS (2009). Predicting control of primary tumor and survival by DCE MRI during early therapy in cervical cancer. Invest Radiol.

[11] Semple SIK, Harry VN, Parkin DE, Gilbert FJ (2009). A combined pharmacokinetic and radiologic assessment of dynamic contrast-enhanced magnetic resonance imaging predicts response to chemoradiation in locally advanced cervical cancer. Int J Radiat Oncol Biol Phys.

[12] Mannelli L, Patterson AJ, Zahra M, Priest AN, Graves MJ, Lomas DJ (2010). Evaluation of nonenhancing tumor fraction assessed by dynamic contrast-enhanced MRI subtraction as a predictor of decrease in tumor volume in response to chemoradiotherapy in advanced cervical cancer. AJR Am J Roentgenol.

[13] Lund KV, Simonsen TG, Hompland T, Kristensen GB, Rofstad EK (2015). Short-term pretreatment DCE-MRI in prediction of outcome in locally advanced cervical cancer. Radiother Oncol.

[14] Lund KV, Simonsen TG, Kristensen GB, Rofstad EK (2017). Pretreatment late-phase DCE-MRI predicts outcome in locally advanced cervix cancer. Acta Oncol.

[15] Brix G, Semmler W, Port R, Schad LR, Layer G, Lorenz WJ (1991). Pharmacokinetic parameters in CNS Gd-DTPA enhanced MR imaging. J Comput Assist Tomogr.

[16] Brix G, Bahner ML, Hoffmann U, Horvath A, Schreiber W (1999). Regional blood flow, capillary permeability, and compartmental volumes: measurement with dynamic CT—initial experience. Radiology..

[17] Tofts PS (1997). Modeling tracer kinetics in dynamic Gd-DTPA MR imaging. J Magn Reson Imaging.

[18] Tofts PS, Brix G, Buckley DL, Evelhoch JL, Henderson E, Knopp MV (1999). Estimating kinetic parameters from dynamic contrast-enhanced *T*_1_-weighted MRI of a diffusable tracer: standardized quantities and symbols. J Magn Reson Imaging.

[19] Sourbron SP, Buckley DL (2013). Classic models for dynamic contrast-enhanced MRI. NMR Biomed.

[20] Lund KV, Simonsen TG, Kristensen GB, Rofstad EK (2019). Pharmacokinetic analysis of DCE-MRI data of locally advanced cervical carcinoma with the Brix model. Acta Oncol.

[21] Hittmair K, Gomiscek G, Langenberger K, Recht M, Imhof H, Kramer J (1994). Method for the quantitative assessment of contrast agent uptake in dynamic contrast-enhanced MRI. Magn Reson Med.

[22] Egeland TAM, Simonsen TG, Gaustad JV, Gulliksrud K, Ellingsen C, Rofstad EK (2009). Dynamic contrast-enhanced magnetic resonance imaging of tumors: preclinical validation of parametric images. Radiat Res.

[23] Green JA, Kirwan JM, Tierney JF, Symonds P, Fresco L, Collingwood M (2001). Survival and recurrence after concomitant chemotherapy and radiotherapy for cancer of the uterine cervix: a systematic review and meta-analysis. Lancet..

[24] Lyng H, Malinen E (2017). Hypoxia in cervical cancer: from biology to imaging. Clin Transl Imaging.

[25] Alongi P, Laudicella R, Desideri I, Chiaravalloti A, Borghetti P, Quartuccio N (2019). Positron emission tomography with computed tomography imaging (PET/CT) for the radiotherapy planning definition of the biological target volume: PART 1. Crit Rev Oncol Hematol.

[26] Fiorentino A, Laudicella R, Ciurlia E, Annunziata S, Lancellotta V, Mapelli P (2019). Positron emission tomography with computed tomography imaging (PET/CT) for the radiotherapy planning definition of the biological target volume: PART 2. Crit Rev Oncol Hematol.

[27] Lee EYP, Hui ESK, Chan KKL, Tse KY, Kwong WK, Chang TY (2015). Relationship between intravoxel incoherent motion diffusion-weighted MRI and dynamic contrast-enhanced MRI in tissue perfusion of cervical cancers. J Magn Reson Imaging.

[28] Dickie BR, Rose CJ, Kershaw LE, Withey SB, Carrington BM, Davidson SE (2017). The prognostic value of dynamic contrast-enhanced MRI contrast agent transfer constant *K*^trans^ in cervical cancer is explained by plasma flow rather than vessel permeability. Br J Cancer.

[29] Hauge A, Wegner CS, Gaustad JV, Simonsen TG, Andersen LMK, Rofstad EK (2017). DCE-MRI of patient-derived xenograft models of uterine cervix carcinoma: associations with parameters of the tumor microenvironment. J Transl Med.

[30] Ellingsen C, Egeland TA, Gulliksrud K, Gaustad JV, Mathiesen B, Rofstad EK (2009). Assessment of hypoxia in human cervical carcinoma xenografts by dynamic contrast-enhanced magnetic resonance imaging. Int J Radiat Oncol Biol Phys.

[31] Hauge A, Gaustad JV, Huang R, Simonsen TG, Wegner CS, Andersen LMK (2019). DCE-MRI and quantitative histology reveal enhanced vessel maturation but impaired perfusion and increased hypoxia in bevacizumab-treated cervical carcinoma. Int J Radiat Oncol Biol Phys.

