# Supplementary material for: DCE-MRI of locally-advanced carcinoma of the uterine cervix: Tofts analysis versus non-model-based analyses
Source: Radiat Oncol. 2020 Apr 15;15:79. doi: 10.1186/s13014-020-01526-2 (PMC7158049; doi:10.1186/s13014-020-01526-2)
Supplement: Supplementary file 1 — Additional file 1. DCE-MRI of locally-advanced carcinoma of the uterine cervix: Tofts analysis versus non-model-based analyses [file 13014_2020_1526_MOESM1_ESM.pdf]

Additional file 1: Figure S1 to:

DCE-MRI of locally-advanced carcinoma of the uterine cervix: Tofts analysis versus non-model-based analyses

Kjersti V. Lund, Trude G. Simonsen, Gunnar B. Kristensen, Einar K. Rofstad

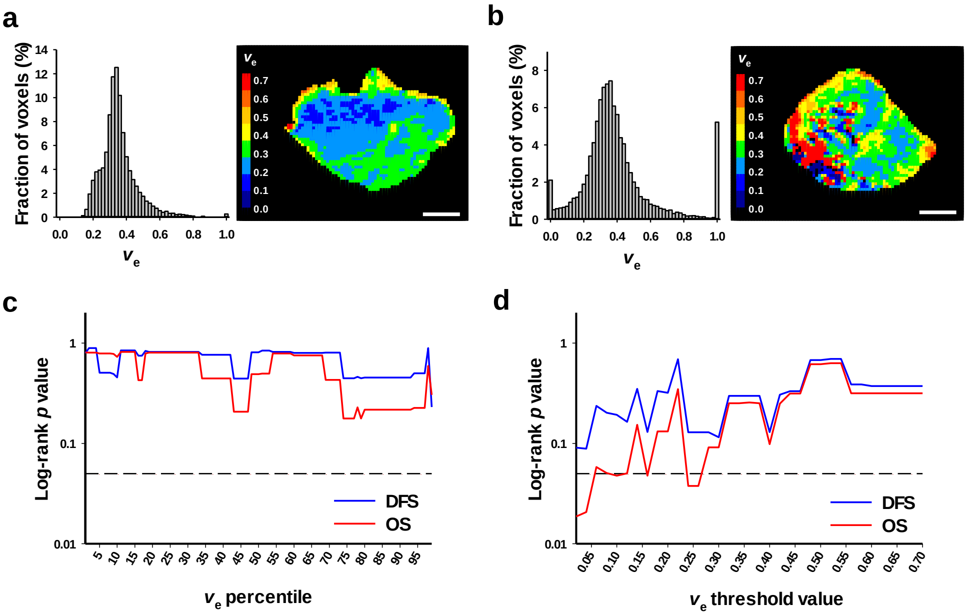

Additional file 1: Figure S1. Analysis of v_e data. Parametric v_e image and v_e frequency distribution of a high-enhancing tumor **a** and a low-enhancing tumor **b**, and plots of log-rank *p* value versus v_e percentile **c** and log-rank *p* value versus v_e threshold value **d**. Scale bars: 1 cm.
